# Long noncoding RNA GAS5 promotes bladder cancer cells apoptosis through inhibiting EZH2 transcription

**DOI:** 10.1038/s41419-018-0264-z

**Published:** 2018-02-14

**Authors:** Miao Wang, Chen Guo, Liang Wang, Gang Luo, Chao Huang, Yawei Li, Dong Liu, Fuqing Zeng, Guosong Jiang, Xingyuan Xiao

**Affiliations:** Department of Urology, Union Hospital, Tongji Medical College, Huazhong University of Science and Technology, Wuhan, 430022 China

## Abstract

Aberrant expression of long noncoding RNA GAS5 in bladder cancer (BC) cells was identified in recent studies. However, the regulatory functions and underlying molecular mechanisms of GAS5 in BC development remain unclear. Here, we confirmed that there was a negative correlation between GAS5 level and bladder tumor clinical stage. Functionally, overexpression of GAS5 reduced cell viability and induced cell apoptosis in T24 and EJ bladder cancer cells. Mechanistically, GAS5 effectively repressed EZH2 transcription by directly interacting with E2F4 and recruiting E2F4 to EZH2 promoter. We previously reported that miR-101 induced the apoptosis of BC cells by inhibiting the expression of EZH2. Interestingly, the present study showed that downregulation of EZH2 by GAS5 resulted in overexpression of miR-101 in T24 and EJ cells. Furthermore, the level of GAS5 was increased under the treatment of Gambogic acid (GA), a promising natural anti-cancer compound, whereas knockdown of GAS5 suppressed the inhibitory effect of GA on cell viability and abolished GA-induced apoptosis in T24 and EJ cells. Taken together, our findings demonstrated a tumor-suppressor role of GAS5 by inhibiting EZH2 on transcriptional level, and additionally provided a novel therapeutic strategy for treating human bladder cancer.

## Introduction

Bladder cancer (BC) is the most common malignancy of the urinary system with an estimated 429,000 new cases and 165,000 deaths annually in the world^1^. Endoscopic resection is generally employed in non-muscle-invasion BC which is often followed by adjuvant chemotherapy. Meanwhile, radical cystectomy is used for treating muscle-invasive BC^2^. However, current primary treatments cannot prevent BC recurrence or progression in high-risk patients. Overall survival of BC remains at a low level, indicating the need for a better knowledge of the molecular basis of BC and exploration of innovative therapeutic strategies.

A major class of newly identified transcripts, long noncoding RNAs (lncRNAs), have been found to drive many important cancer phenotypes^3^. Accumulating studies demonstrated that lncRNAs regulated proliferation, metastasis and apoptosis of tumor cells^4,5^. Out of numerous cancer-related LncRNAs, growth arrest-specific 5 (GAS5), whose gene is located at chromosome 1q25.1, plays an essential role in the regulation of cancer cell survival^6^. Previous studies reported that GAS5 lowly expressed in various neoplasm (e.g., skin cancer^7^, breast cancer^8^ and bladder cancer^9^) and was associated with cell cycle arrest and apoptosis of tumor cells^9^. Recently, Zhang et al found that overexpression of GAS5 promoted apoptosis in drug-resistant BC^10^. These findings indicated a potential role for GAS5 in BC. However, the underlying molecular mechanisms remain unknown, and the expression of GAS5 after the treatment with anti-cancer agent in BC cells has not been investigated.

LncRNAs had been demonstrated to interact with the polycomb repressive complex 2 (PRC2) to reprogram chromatin state and regulate cancer invasiveness and metastasis^11,12^. The enhancer of zeste homolog 2 (EZH2), as a constitution of PRC2, is a histone 3 lysine 27 (H3K27) methyltransferase^13^. EZH2 is ectopically expressed in BC cells to facilitate BC development and progression^9,10^. Our previous work showed that inhibition of EZH2 could promote BC cell apoptosis after the treatment with Gambogic acid (GA), a natural plant ingredient^14^. It has been well acknowledged that LncRNAs regulated the development of BC though the regulatory interactions with EZH2. Upregulated lncRNA H19 promoted BC cell metastasis by increasing the binding of EZH2 and H3K27me3 with the Nkd1 promoter^15^. LncRNA HOTAIR regulated metastatic progression by associating with PRC2 subunits SUZ12 or/and EZH2^11^. However, whether lncRNAs participate the regulation of the transcription of EZH2 in BC has yet to be studied.

In this study, we showed that GAS5 expression level was negatively related to clinical stage of BC. GAS5 inhibited EZH2 transcription by interacting with transcription factor E2F4 to promote BC cells apoptosis. Moreover, GAS5 acted as a key factor in GA-induced apoptosis of BC cells.

## Results

### GAS5 was lowly expressed in human BC tissues

**Fig. 1:**
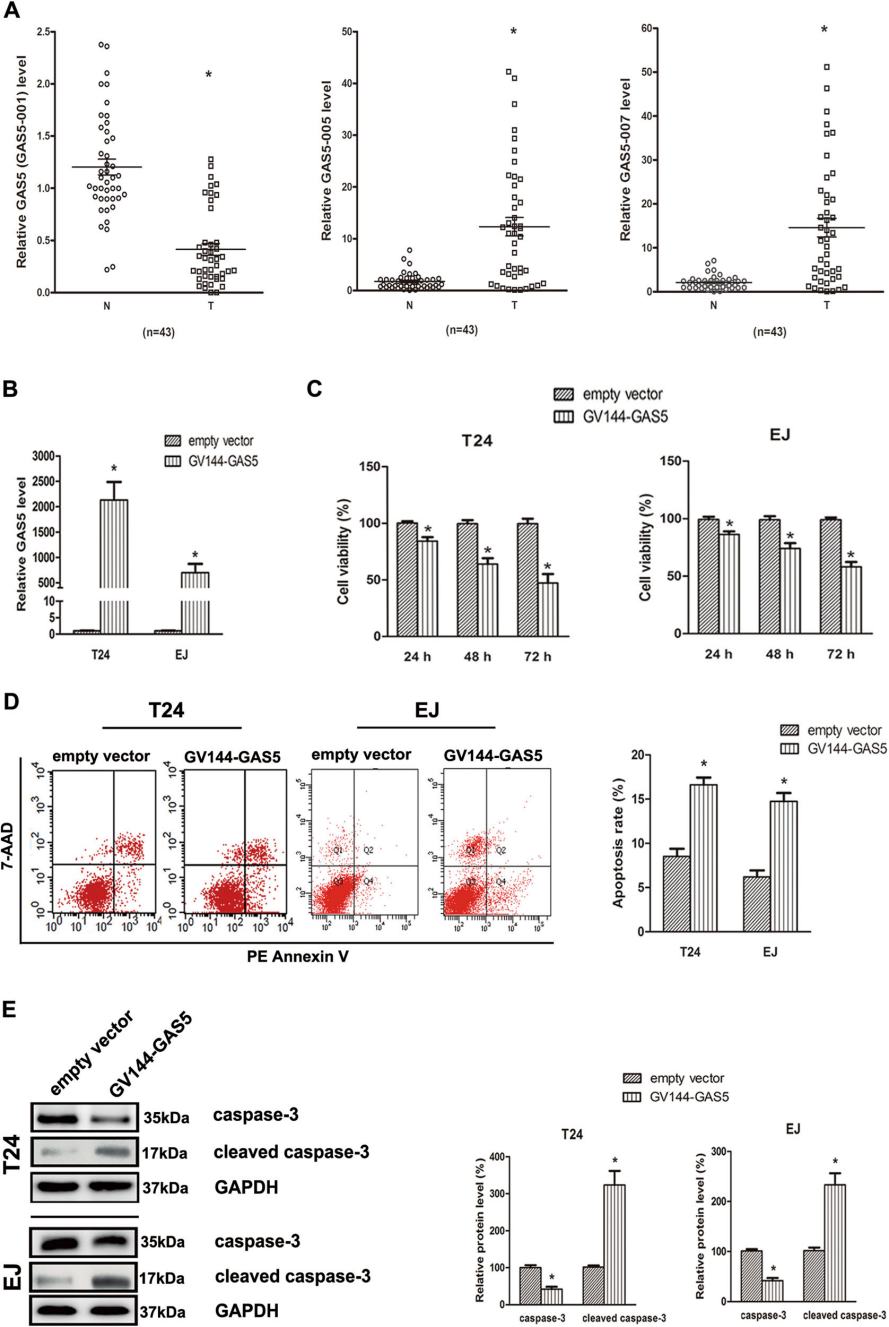
GAS5 is lowly expressed in human BC tissues, overexpression of GAS5 suppressed viability and promoted apoptosis of BC cells. **a** Total RNA was extracted from human normal bladder (N) and the paired tumor (T) tissues of 43 patients, diagnosed with different stages of bladder cancer. GAS5 (GAS5-001), GAS5-005, and GAS5-007 expression levels were then detected by RT-qPCR assay. **b** GV144-GAS5 or the empty GV144 vector plasmids were transfected into BC cells. Forty eight hour later, overexpression efficient of GAS5 was evaluated by RT-qPCR. **c** GV144-GAS5 or the empty vector was transfected into BC cells for 24 h, 48 h, 72 h respectively, and cell viabilities were assessed by MTT assay. **d** Forty eight hour after transfection of GV144-GAS5 or the empty vector, cells were harvested and stained by Annexin V-PE and 7-AAD, and apoptosis rates were analyzed by flow cytometry. **e** Seventy two hour after transfection of GV144-GAS5 or the empty vector, total protein was extracted, and caspase-3 and cleaved caspase-3 protein levels were then assessed by Western-blot assay, GAPDH was served as the internal control. *P < 0.05 versus control groups

LncRNA GAS5 (GAS5-001) and other two isoforms (GAS5-005 and GAS5-007) were studied here. We evaluated the expression levels of different transcripts of GAS5 in 43 pairs of bladder urothelial carcinoma samples and their adjacent normal bladder tissues from BC patients at different stages. A significant downregulation of GAS5 (GAS5-001) level was observed in 33 out of 43 (76.7%) BC tissues, with the overall mean level of GAS5 (GAS5-001) 3-fold lower than that of the adjacent normal bladder tissues. By contrast, the levels of GAS5-005 and GAS5-007 were significantly increased in BC tissues (Fig. 1a). Our findings are consistent with previous study that GAS5 (GAS5-001) acts as a tumor-suppressive GAS5 transcript, and GAS5-007 is an oncogenic GAS5 transcript in some other tumor types^16^. GAS5 (GAS5-001) was single out for further studies. As shown in Table 1, a distinct relationship was found between low GAS5 expression and invasive potential of BC (*P* < 0.001). The data highlights the role of GAS5 as a crucial cancer-suppressor in BC.

**Table 1:** Correlation between GAS5 expression and clinicopathological factors

Factors	Cases	Expression of GAS5		*P*-value
		Low	High	
Overall	43	33	10	
Gender				
Male	31	24	7	1
Female	12	9	3	
Age				
≤55	7	5	2	0.656
>55	36	28	8	
Stage				
Primary tumor				
NMI (Ta+Tis+TI)	22	13	9	0.009
MI (T2+T3+T4)	21	20	1	
Lymph node metastasis				
Absent (N0)	14	11	3	1
Present (N1+N2+N3)	29	22	7	
Distant metastasis				
Absent (N0)	40	31	9	0.558
Present (M1)	3	2	1	
Grade				
Low grade	13	9	4	0.458
High grade	30	24	6	

*NMI* non-muscle invasive, *MI* muscle invasive

### Upregulation of GAS5 suppressed viability and promoted apoptosis of BC cells in vitro

Given that GAS5 is downregulated in BC tissues in our study, we further explored the effects of GAS5 on BC cell biological activity. GV144 and GV144-GAS5 were transfected into T24 and EJ cells, the mRNA expression levels of GAS5 were significantly increased in both T24 and EJ cells after transfection of GV144-GAS5 (Fig. 1b). MTT assays were performed and results showed that transfection of GV144-GAS5 significantly decreased cell viabilities of T24 and EJ cells in a time-dependent manner (Fig. 1c). On the other hand, flow cytometric assays were performed to study the effect of GAS5 on the apoptosis of BC cell lines. Forced expression of GAS5 promoted cell apoptosis rates in T24 and EJ cells (Fig. 1d). Consistently, 72 h after transfection of GV144-GAS5, Western-blot assays were performed and apoptotic executive protein caspase-3 was found to be activated in both BC cell lines (Fig. 1e).

### Knockdown of GAS5 inhibited GA-induced apoptosis of BC cells

**Fig. 2:**
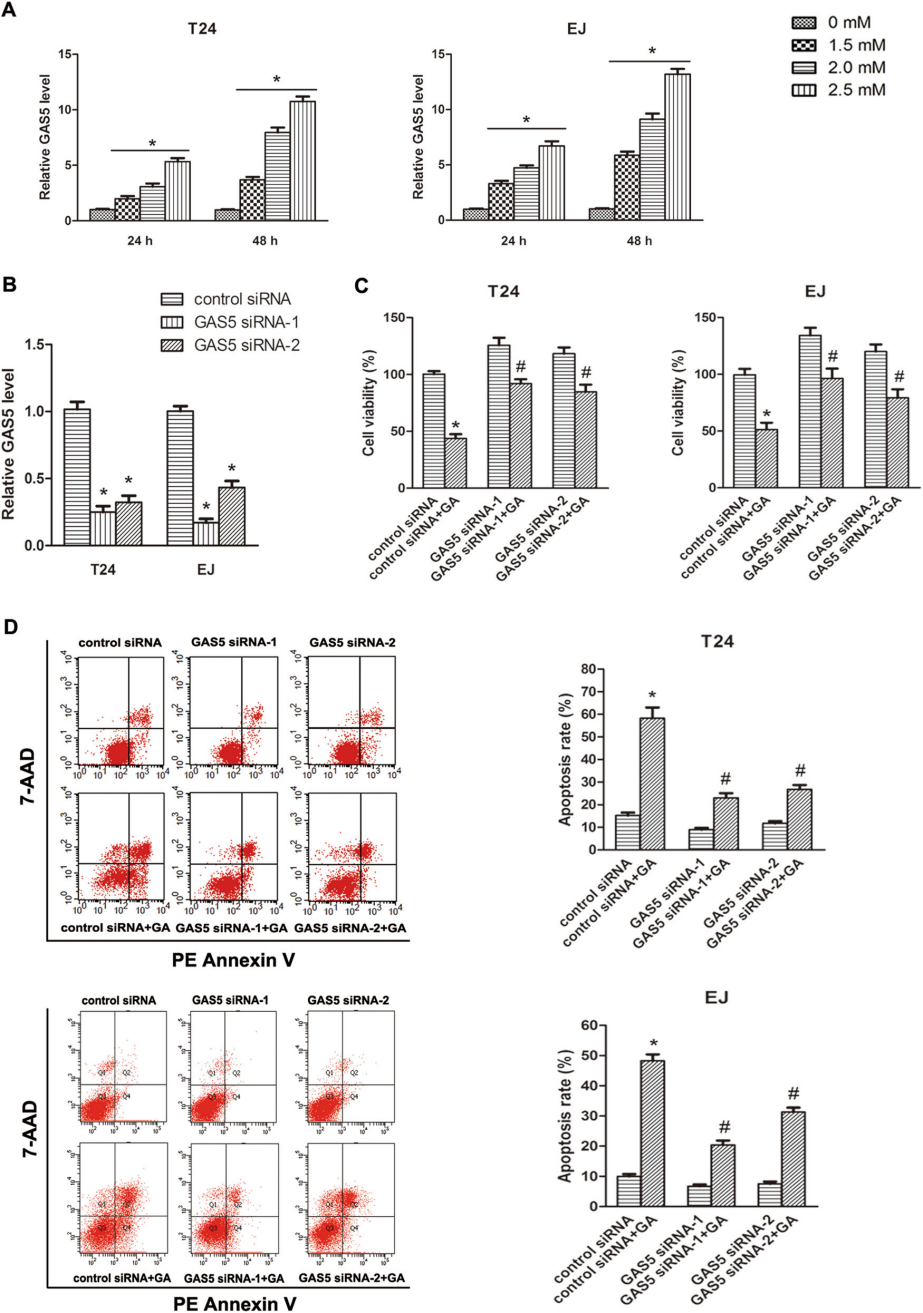
GA enhanced GAS5 expression, and knockdown of GAS5 suppressed GA-induced apoptosis of BC cells. **a** T24 and EJ cells were treated with different concentrations of GA (0, 1.5, 2, 2.5 μM) for 24 or 48 h, and GAS5 expression levels were assessed by RT-qPCR. **b** The expression levels of GAS5 in T24 and EJ cells were detected by RT-qPCR assay, with transfection of GAS5 siRNA-1, -2 or the control siRNA for 48 h. **c** Forty eight hour after transfection of siRNAs, 2.5 μM GA or isopyknic PBS was treated. Twenty four hour later, cells were collected and cell viabilities were evaluated by MTT assay. **d** Meanwhile, cell apoptosis rates were analyzed by flow cytometry analyzed. Results were presented as the means ± SD of triplicates. *P < 0.05 versus control groups, ^#^a significant difference between GAS5 siRNA and control siRNA groups

GA is increasingly recognized as novel and promising natural anti-cancer compound for BC^14,17,18^. Here, we investigated the potential role of GAS5 in anti-cancer compound GA-induced apoptosis of BC cells. Both T24 and EJ cells were treated with different concentrations of GA (0, 1.5, 2, 2.5 μM) for 24 h or 48 h respectively, we found GAS5 expressions were significantly increased by the treatment of GA in a both time-dependent and dose-dependent manner (Fig. 2a). Next, we explored whether GAS5 affected GA-induced apoptosis of BC cells. GAS5-siRNA-1 and GAS5-siRNA-2 were transfected into BC cells, and effectively suppressed GAS5 levels (Fig. 2b). MTT assays showed that knockdown of GAS5 reversed GA-induced inhibition on cell viability (Fig. 2c). Flow cytometric assays revealed that knockdown of GAS5 repressed GA-induced cell apoptosis (Fig. 2d). These observations demonstrated that GAS5 was a crucial factor in the anti-cancer effect of GA in BC cells, which also suggested that GAS5 is an attractive chemotherapeutic target in future BC treatment.

### Upregulation of GAS5 inhibited EZH2 transcription in BC cells

**Fig. 3:**
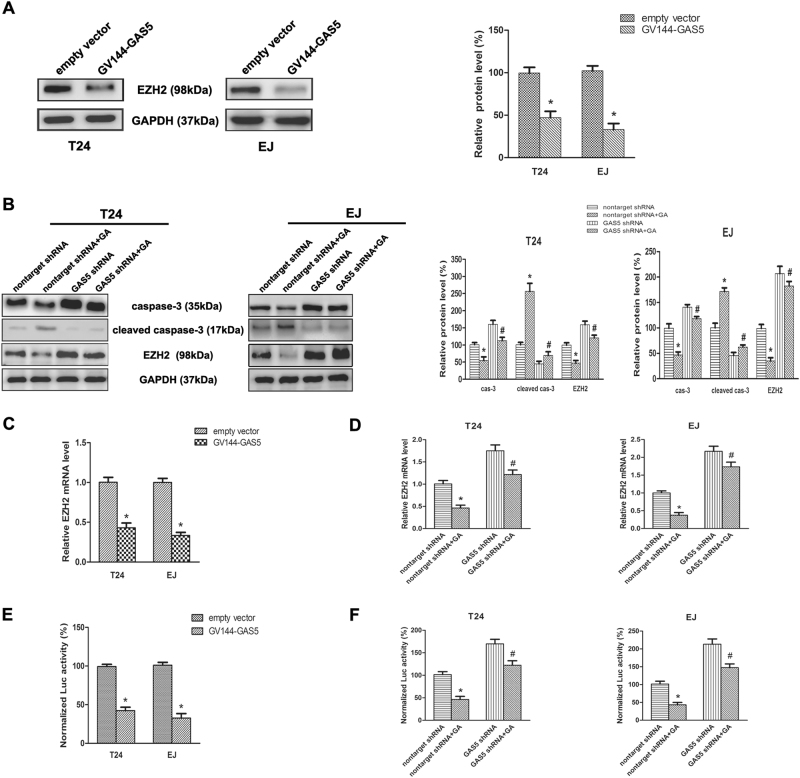
GAS5 inhibited transcriptional of EZH2, and knockdown of GAS5 suppressed GA-induced inhibition of EZH2 transcription. **a** GV144-GAS5 or the empty vector was stably transfected into BC cells, EZH2 protein levels were detected by Western-blot assay. **b** GAS5 shRNA or the control (nontarget shRNA) was stably transfected into BC cells, and 2.0 μM GA or isopyknic PBS was treated for 48 h, the Western-blot assay indicated that GAS5 shRNA evidently inhibited GA-induced downregulation of EZH2 protein expression and the activation of caspase-3. **c** BC cells stably transfected with GV144-GAS5 or the empty vector were collected, and EZH2 mRNA levels were assessed by RT-qPCR assay. **d** BC cells stably transfected with GAS5 shRNA or the control shRNA (nontarget shRNA) were treated with 2.0 μM GA or isopyknic PBS for 48 h, RT-qPCR showed that GAS5 shRNA remarkably reversed the inhibition of EZH2 mRNA expression by GA. **e** EZH2 transcriptional activities of BC cells stably transfected with GV144-GAS5 or the empty vector were assessed by luciferase reporter assay. **f** The cells were then treated with 2.0 μM GA or isopyknic PBS. Forty hours later, EZH2 transcriptional activities were analyzed, the activity of firefly luciferase was normalized by renilla luciferase. Results were presented as the means ± SD of triplicates. *P < 0.05 versus controls, ^#^a significant difference between GAS5 shRNA and nontarget shRNA groups

Our previous work and several other studies have demonstrated that EZH2 is an essential factor in regulating cell biological activity in BC^14,19,20^. Additionally, both EZH2 and GAS5 have involved GA-induced BC cell apoptosis^14^. Therefore, we investigated whether GAS5 influences the expression of EZH2. GV144-GAS5 was stably transfected into T24 and EJ cells, and forced expression of GAS5 markedly downregulated EZH2 protein level (Fig. 3a). GAS5 shRNA were subsequently transfected into EJ and T24 cells. As shown in Fig. 3b, transfection of GAS5 shRNA reversed GA-induced downregulation of EZH2 protein and the activation of caspase-3, when compared with negative control. Accordingly, forced expression of GAS5 restrained EZH2 mRNA expression (Fig. 3c), and knockdown of GAS5 restored GA-induced suppression of EZH2 mRNA expression in T24 and EJ cells (Fig. 3d). To illustrate the mechanism of regulation of EZH2 expression by GAS5, human EZH2 luciferase reporter plasmid was used for luciferase reporter assay. Forced expression of GAS5 resulted in a decreased promoter activity of EZH2 in EJ and T24 cells (Fig. 3e). Meanwhile, knockdown of GAS5 reversed the downregulation of transcriptional activity of EZH2 mRNA which was induced by GA treatment (Fig. 3f). These results proved that GAS5 negatively regulated EZH2 transcription in BC.

### GAS5 downregulated transcriptional expression of EZH2 through recruitment of E2F4 in BC cells

**Fig. 4:**
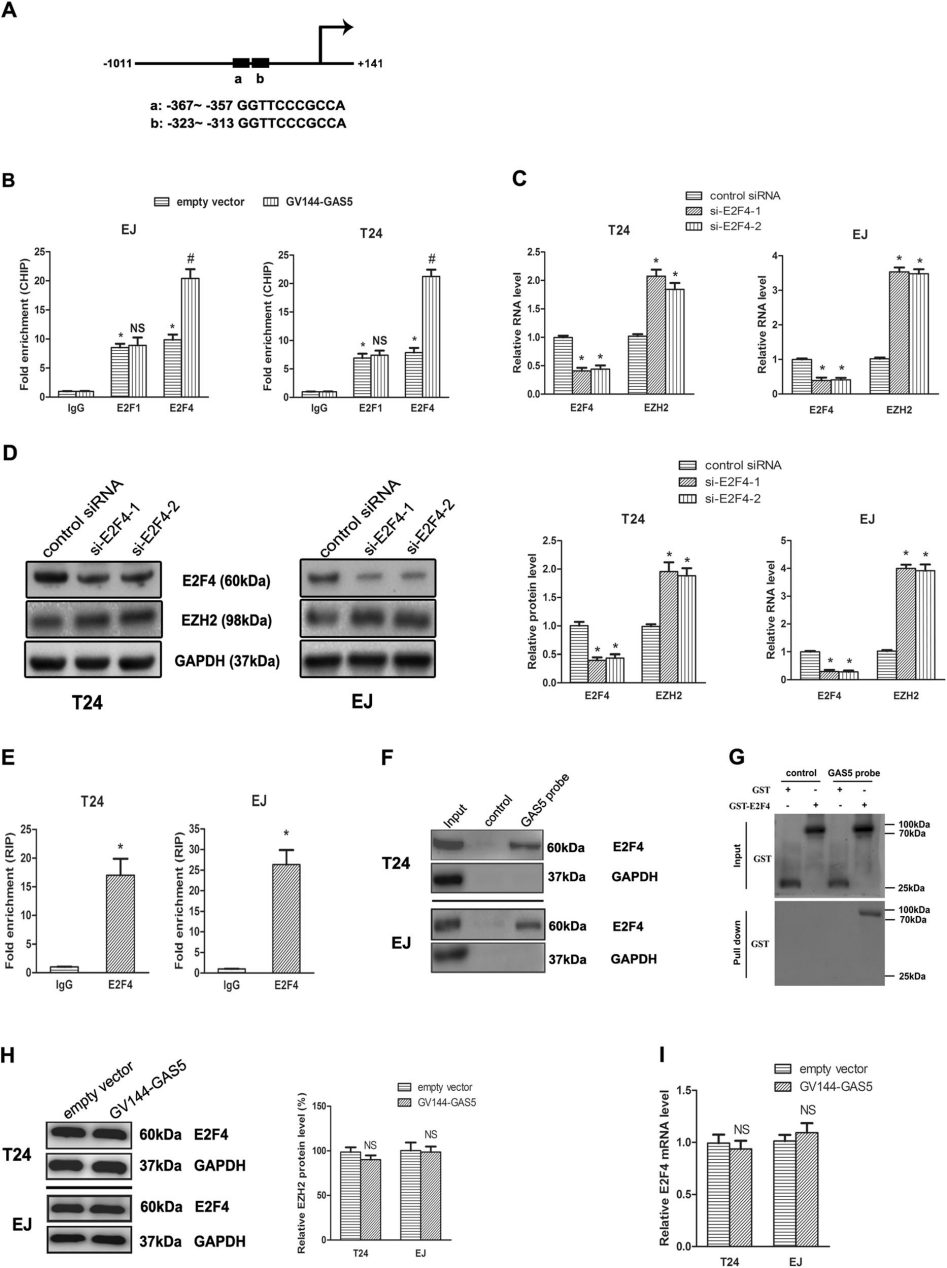
GAS5 bind and recruited transcription factor E2F4 to EZH2 promoter to inhibited EZH2 transcription. **a** E2Fs binding sites in EZH2 promoter: a, b represents putative binding sites, +1 represents the most 5′ nucleotide in the longest identified cDNA published in the NCBI database. **b** GV144-GAS5 or the empty vector was transfected into BC cells, and chrome immunoprecipitations were performed by using specific anti-E2F1 or anti-E2F4 antibodies. **c** E2F4 siRNAs (E2F4-1, 2) or the control siRNA were transfected into BC cells for 48 h, E2F4 and EZH2 mRNA levels were then assessed by RT-qPCR. **d** BC cells transfected with E2F4 siRNAs or the control siRNA for 72 h were collected, and EZH2 and E2F4 protein levels were detected by Western-blot assay. **e** RNA immunoprecipitations were performed in BC cells, and the relative quantities of GAS5 were detected by RT-qPCR assay, normalized to the input groups. IgG and E2F4 represented for the groups coprecipitation with IgG protein and anti-E2F4 antibody respectively. **f** Total proteins were extracted from T24 and EJ cells, and then lncRNA GAS5 pull-down assay was performed. The E2F4 protein levels were evaluated by Western-blot. GAS5 probe represented the biotin-labeled GAS5 probe group and control stood for the oligo probe group. **g** GST and GST-E2F4 fusion proteins were applied for lncRNA GAS5 pull-down assay, and the GST protein levels were analyzed by Western-blot. **h**, **i** BC cells with GV144-GAS5 or the empty vector was harvested, and expression levels of E2F4 protein (**h**) and E2F4 mRNA (**i**) were detected. *P < 0.05 versus the control groups, NS no significant difference between the groups, ^#^a significant difference between GV144-GAS5 and empty vector groups

EZH2 transcription is regulated by several transcription factors such as E2Fs^21,22^. We found that there were two E2F-binding sites in EZH2 promoters (Fig. 4a). The E2F family is mainly comprised of 5 members (E2F1-5). Though both E2F1 and E2F4 could bind to EZH2 promoter, the CHIP assay showed that only E2F4’s binding capacity was improved evidently with GAS5 overexpression (Fig. 4b). In addition, E2F4 is the most abundant of all E2Fs, and E2F4-loss plays a key role in suppressing bladder carcinogenesis^23^. Therefore, E2F4 was chosen as the object of our study. E2F4 was then knockdown by siRNA, and the results of RT-qPCR and western blot revealed that EZH2 expression was significantly upregulated (Fig. 4c, d). Following that, RNA immunoprecipitation (RIP) assay was performed, which demonstrated that E2F4 protein could bind to GAS5 (Fig. 4e). The direct interaction between E2F4 and GAS5 was further verified by applying total protein (Fig. 4f) and GST-E2F4 fusion protein (Fig. 4g) to GAS5 pull-down assay. In addition, we found GAS5 did not impact the expression of E2F4 protein and E2F4 mRNA (Fig. 4h, i). Taken together, we concluded that GAS5 inhibited EZH2 transcriptional activity by recruiting transcription factor E2F4 to EZH2 promoter.

### Knockdown of EZH2 improved miR-101 transcriptional activity and overexpression of EZH2 suppressed GA-stimulated upregulation of miR-101

**Fig. 5:**
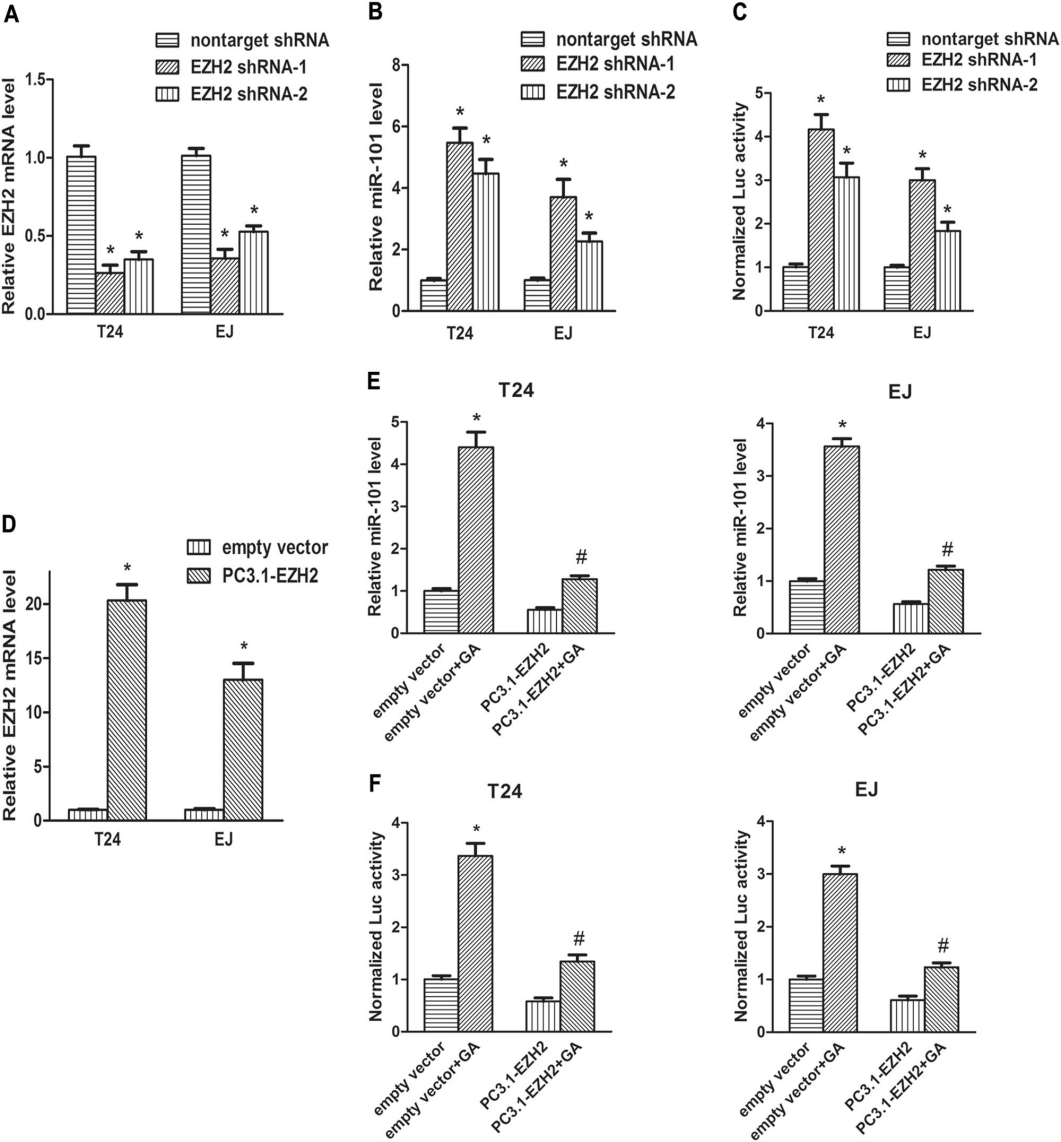
Knockdown of EZH2 improved miR-101 transcription, and overexpression of EZH2 suppressed GA-stimulated upregulation of miR-101. **a**–**c** EZH2 shRNA was stably transfected into BC cells, RT-qPCR was performed to assess EZH2 mRNA (**a**) and miR-101 (**b**) expression, luciferase reporter assay was performed to detect miR-101 transcriptional activity (**c**). **d** BC cells stably transfected with PC3.1-EZH2 or the empty PC3.1 vector was harvested and the transfection efficiency was evaluated. **e**, **f** Then, 2.0 μM GA or isopyknic PBS was added. Forty eight hour later, miR-101 expression levels were assessed (**e**), and 40 h later, miR-101 transcriptional activities were detected (**f**). *P < 0.05 versus controls, ^#^a significant difference between PC3.1-EZH2 and empty vector groups

It is generally recognized that miR-101 inhibits EZH2 by directly targeting to its 3’-UTR^24^. But a recent study discovered that EZH2 suppressed miR-101 transcription reversely in hepatocellular carcinoma^25^. We sought out to examine this theory in BC. We blocked EZH2 expression in T24 and EJ cells by stable transfection of EZH2 shRNA (Fig. 5a), subsequently RT-qPCR indicated that miR-101 expression was upregulated (Fig. 5b). To verify the effect of EZH2 on miR-101 transcription, human miR-101-2 reporter plasmid was transfected into T24 and EJ cells for luciferase reporter assay. It indicated that transcriptional activity of miR-101 was significantly increased by knockdown of EZH2 (Fig. 5c). The results suggested that downregulation of EZH2 enhanced miR-101 expression by improving miR-101 transcriptional activity.

Next, EZH2 was stably overexpressed in T24 and EJ cells by transfection of PC3.1-EZH2 (Fig. 5d). 2.0 μM GA or equal volume of PBS was then treated. Forty eight hours later, RT-qPCR revealed that GA upregulated miR-101 expression. However, that effect was significantly suppressed by overexpression of EZH2 (Fig. 5e). Consistently, similar results were observed by luciferase reporter assay (Fig. 5f). These results suggested that overexpression of EZH2 restored GA-induced upregulation of miR-101.

### Knockdown of GAS5 suppressed GA-induced growth inhibition of BC in vivo

**Fig. 6:**
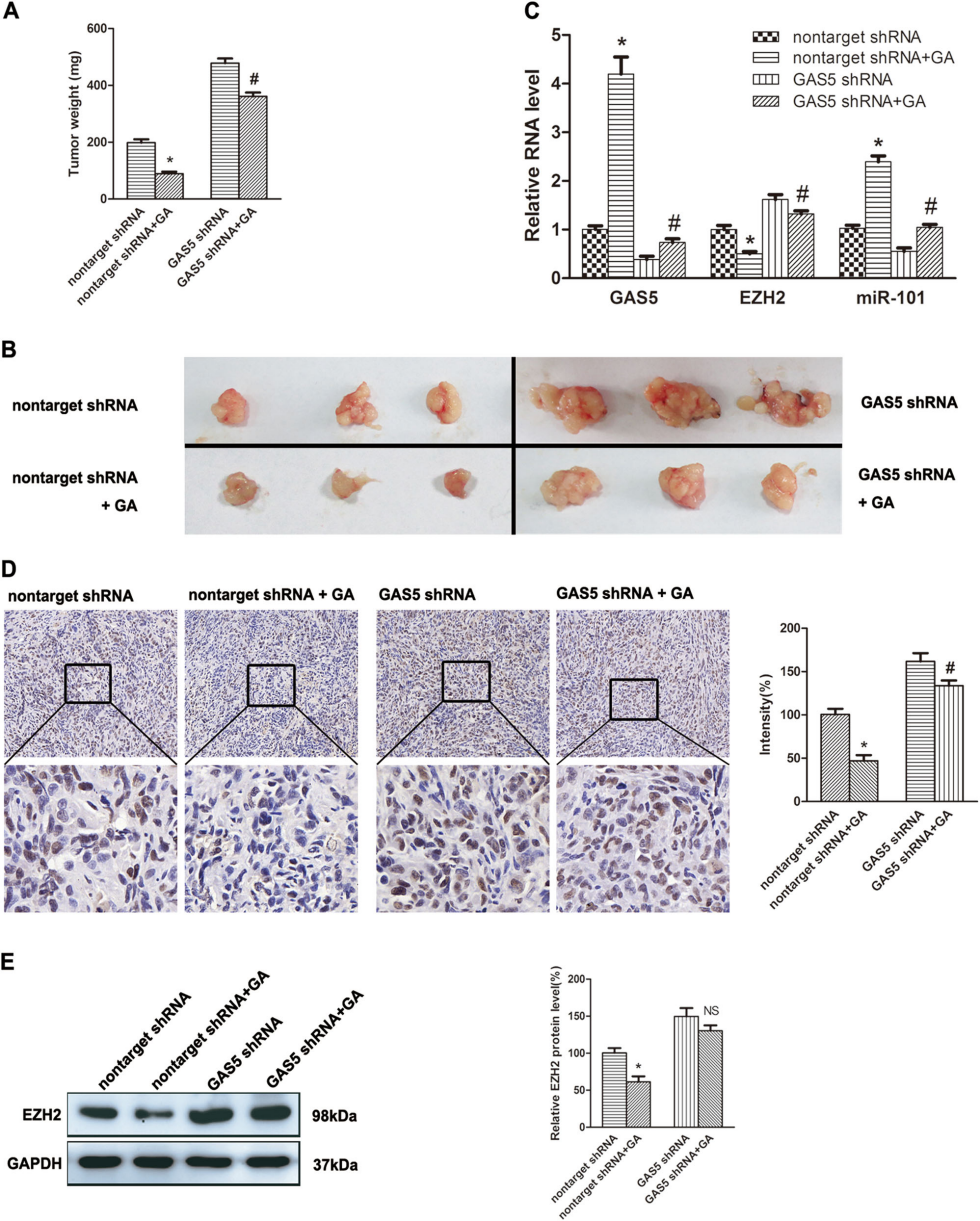
Knockdown of GAS5 inhibited GA-induced apoptosis of BC cells in vivo. Four-week-old Balb-c nude mice were randomly divided into two groups, and EJ cells (1 × 10^6^) with GAS5 shRNA or the nontarget shRNA stable transfection were injected s.c. respectively. Two weeks later, GA (1.8 mg kg^−1^) or the vehicle was administered i.v. once every other day. Sixteen days after that, the mice were sacrificed and the tumors were removed, weighed (**a**), and photographed (**b**). **c** Then, total RNA was extracted from the tumor tissues, and expressions of GAS5, EZH2 mRNA and miR-101 were detected by RT-qPCR (**c**). **d** Immunohistochemistry analysis of EZH2 was obtained from tumors (100×, 400×). **e** Total protein was extracted and expression of EZH2 protein was assessed by Western-blot assay. *P < 0.05 versus controls, ^#^a significant difference between GAS5 shRNA and nontarget shRNA groups

Finally, we assessed the effects of GAS5 on bladder xenograft tumors and its effect on GA-induced growth inhibition of bladder xenograft tumor. The tumors were removed, weighed (Fig. 6a) and photographed (Fig. 6b). It is obvious that tumor growth was inhibited by GA and promoted by knockdown of GAS5. The inhibitive effect of GA was reversed by knockdown of GAS5. Total RNA was extracted from the tumor tissues and RT-qPCR illustrated that GA decreased EZH2 mRNA expression and increased miR-101 expression, but these effects were significantly suppressed by knockdown of GAS5 (Fig. 6c). Moreover, immunohistochemistry and Western-blot indicated that GA-induced downregulation of EZH2 protein was abolished by knockdown of GAS5 (Fig. 6d, e).

## Discussion

Increasing data proved that lncRNAs participated in BC’s formation, progression, and metastasis^26^. The human H19 gene is the first lncRNA that is found to be oncogene in the bladder, which is rarely expressed in adult tissue, but highly expressed in BC^27^. Since then, emerging lncRNAs such as MALAT1, TUG1, and UCA1 had been demonstrated to be tumor markers or prognostic indicators of BC^26^. Meanwhile, it has been previously demonstrated that downregulated lncRNA MEG3 activates autophagy and increases cell proliferation in BC^28^. Forced expression of MEG3 inhibits tumor cell proliferation and colony formation by inducing accumulation of p53 protein^29^. Here, we confirmed that lncRNA-GAS5 was lowly expressed in BC tissues, and further demonstrated that lower GAS5 levels were associated with poor prognosis in BC. It has been reported that GAS5 inhibited cell proliferation by regulating the expression of CDK6 and CCL1 in BC^9,30^. In the present study, our results showed that upregulation of GAS5 promoted apoptosis of BC cells. Furthermore, we found that GAS5 inhibited EZH2 transcription by recruiting transcription factor E2F4 to EZH2 promoter, and suppression of GAS5 reversed the growth inhibition effects of GA on BC both in vivo and in vitro. These findings shed new light on a novel tumor-suppressing mechanism for GAS5 in BC.

Although the function of the vast majority of lncRNAs remains to be identified, it is demonstrated that several lncRNAs can regulate the binding of transcription factors or transcriptional repressors to gene promoters and thereby cause gene activation or silencing^31,32^. Previous study also found that GAS5 bound to transcription factor GR (glucocorticoid receptor) and titrated it away from its target gene promoters^33^. RNA-seq data showed several lncRNAs regulated cell cycle gene expression by targeting E2F transcription factors in breast cancer^34^. E2Fs are frequently amplified and overexpressed in numerous cancers, and the activation of E2Fs is an important molecular feature of human BC^20^. E2F activity is regulated by pocket proteins (Rb, p107 and p130), HDAC and Brg, which bind to E2Fs in their active under-phosphorylated form, and inhibit the transcription of E2Fs target genes^35^. Our latest research demonstrated GAS5 interacted with E2F1 and enhanced the binding of E2F1 to the promoter of P27Kip1, which is a known regulator of cell cycle^36^. Here, we explored the role of E2Fs associated with GAS5 in BC. Our data showed GAS5 could directly interact with E2F4. ChIP assay showed that GAS5 enhanced the binding of E2F4 to EZH2 promoter. Thus, we concluded that by recruitment of E2F4 to EZH2 promoter, upregulated GAS5 inhibited transcription of EZH2. Our research further develops the understanding of the regulatory function of GAS5 on the transcription of downstream genes in BC.

EZH2 is a histone methyltransferase component of the PRC2. Both gain- and loss-of-function mutations of EZH2 have been involved in tumorigenesis^37,38^. Though downregulation and loss-of-function mutations suggest tumor suppressive activity of EZH2 in some cancers, evidence to demonstrate that EZH2 with gain-of-function mutations act as oncogene in numerous others, such as prostate cancer, breast cancer and BC^37^. Previously, we demonstrated that BRD4 facilitated EZH2 transcription through the recruitment of C-MYC to EZH2 promoter^19^. The E2Fs also have been reported to manipulate EZH2 transcription in several malignancies including BC^39^. Rb/RB1 phosphorylation activated the expression of EZH2 through direct binding of E2F with the EZH2 promoter in BC and small cell lung cancer^20,40^. Present research showed that overexpression of GAS5 promoted BC cells apoptosis by inhibiting transcription of EZH2, and transcriptional inhibition of EZH2 resulted from the recruitment of E2F4 to EZH2 promoter by GAS5. These findings indicated the bidirectional regulation of the transcription of EZH2 by E2Fs in BC, consistent with the findings of an earlier study which reported that E2Fs are divided into two subgroups based on their transcriptional properties: activating (E2F1-3) and repressing (E2F4-5) role of E2Fs^41^. Our results expanded our knowledge of the regulatory network between E2Fs and EZH2 in BC, and confirmed the key role of E2F4 in BC development.

Our prior recent work had revealed the regulation of EZH2 at the posttranscriptional level by miR-101^14^. miRNAs mostly bind to the 3′-UTR of mRNAs, and give rise to target mRNAs destabilization and/or translational inhibition^24^. miR-101 has been regarded as tumor-suppressive miRNA, and it could directly target 3’-UTR of EZH2 mRNA and inhibit proliferation, migration, and angiogenesis of tumor cells^42^. Interestingly, previous study has also showed EZH2 inhibited miR-101 transcription in hepatocellular carcinoma^43^. In our present study, we found that EZH2 negatively regulated miR-101 transcription in BC. Our findings add to the accumulating evidence that suggests there is a positive feedback loop between EZH2 and miR-101.

Various EZH2 inhibitors had been developed, but none of them have been applied in clinical practice for BC patients^37,44^. Gambogic acid (GA, C38H44O8), derived from the resin of Garcinia, has long been used as antioxidant, antiviral, anti-inflammatory and parasiticide medicine^45^. Recent researches found that GA was cytotoxic to tumor cells but had minimal toxicity to normal cells^46,47^. Thus, GA is a potential chemotherapeutic agent for clinical application, and it has already finished a phase II clinical trial in China^48^. We previously reported that GA induced BC cell apoptosis through inhibiting the expression of EZH2^14^. In this study, we found GA positively regulating GAS5 expression, and the pro-apoptotic effect of GA was repressed by GAS5 knockdown both in vivo and in vitro. These results support the feasibility of using GAS5 to act as a promising target for development of novel anti-cancer agents in BC.

**Fig. 7:**
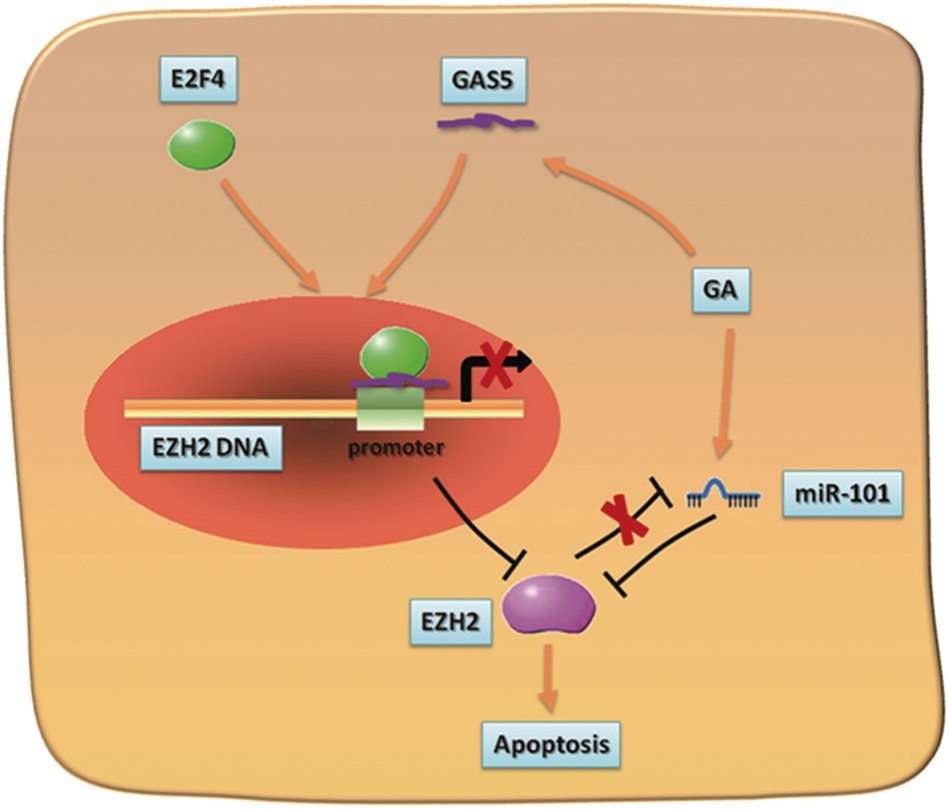
A hypothetical working model of the role of GAS5 in bladder cancer apoptosis. Overexpression of GAS5 effectively increases the binding of E2F4 to EZH2 mRNA promoter, resulting in repression of EZH2 transcription that promotes tumor cell apoptosis. Meanwhile, this study shows that EZH2 negatively regulated miR-101 transcription in BC, and as we known, miR-101 is a repressor of EZH2. Thus, upregulation of GAS5 results in a positive feedback loop between EZH2 and miR-101. Furthermore, Gambogic acid (GA), a promising natural anticancer compound, could induce bladder cancer cell apoptosis though regulating the expression of both GAS5 and miR-101

In conclusion, we first identified lncRNA GAS5 as an important prognosis maker for BC patients. Most significantly, this study then demonstrated a novel regulatory function of GAS5 on the transcriptional level of EZH2 by enhancing the binding of E2F4 to EZH2 promoter (Fig. 7). Since the interaction between GAS5 and EZH2 plays an essential role in the cell apoptosis of BC, our findings demonstrate the possibility of using GAS5 as therapeutic target for treatment of BC, and extend existing knowledge about the molecular mechanism that underlies BC progression.

## Materials and methods

### Reagents

GA and DMSO were purchased from Sigma (St Louis, MO, USA). GA was dissolved in DMSO at a concentration of 25 mmol/L and was stored at -20 °C. For in vitro study, GA was then dissolved in culture medium. For in vivo study, GA was then dissolved in 0.9% NaCl.

### Patients and specimens

43 patients who had undergone radical cystectomy at Department of Urology, Union Hospital of Tongji Medical College from August 2012 to May 2014 were selected for this study. All the patients were diagnosed as transitional cell cancer by histopathologic biopsy, but none of the patients received anti-cancer therapy before surgical resection. Both carcinoma tissues and adjacent non-carcinoma tissues (at least 2 cm from the tumor site) were collected and evaluated by at least two experienced pathologists. All the specimens were staged according to TNM Classification of Malignant Tumours—7th edition, and graded according to the 2004 World Health Organization Consensus Classification and Staging System for bladder neoplasms. A summary of clinical data is listed in Table 1. The research was approved by the Institutional Review Board of Tongji Medical College of Huazhong University of Science and Technology, and informed consent was obtained from each patient before surgery.

### Cell culture

The human bladder cancer cell lines T24 and EJ were maintained by our laboratory. Cells were cultured in RPMI-1640 medium (Gibco, Grand Island, NY, USA), supplemented with 10% FBS (HyClone, Logan, UT, USA), 100 U mL^−1^ penicillinand and 100 μg mL^−1^ streptomycin (Gibco). All cells were cultured in a humidified atmosphere, at 37 °C with 5% CO_2_.

### Real-time quantitative Polymerase Chain Reaction (RT-qPCR)

The relative quantity of lncRNA and mRNA were measured by RT-qPCR, and had been described as previously^14^. The gene-specific primers were designed by Primer Premier 5.0 software (Premier Biosoft International, Palo Alto, CA, USA), and the sequences are list in Table 2. GAPDH served as internal control.

**Table 2:** Sequences in the study

Oligonucleotides	Sequence (5′–3′)
Primers	
GAS5 forward	ACAGGCATTAGACAGAAAGC
GAS5 reverse	TACCCAAGCAAGTCATCCA
GAS5-005 forward	TGAGGTATGGTGCTGGGTG
GAS5-005 reverse	GAGGGGAGAGAAGCACTAACA
GAS5-007 forward	CTGTGAGGTATGGTGCTGG
GAS5-007 reverse	GTGCCGTAGGAAGTTTGC
EZH2 forward	CCCTGACCTCTGTCTTACTTGTGGA
EZH2 reverse	ACGTCAGATGGTGCCAGCAATA
E2F4 forward	CGGCGGATTTACGACATTA
E2F4 reverse	CTTGTGCTGGTCTAGTTCTTGC
GAPDH forward	GAAGGTGAAGGTCGGAGTC
GAPDH reverse	GAAGATGGTGATGGGATTTC
siRNA/shRNA	
GAS5 siRNA-1 sense	GGACCAGCTTAATGGTTCT
GAS5 siRNA-1 anti-sense	AGAACCATTAAGCTGGTCC
GAS5 siRNA-2 sense	GCAAGCCTAACTCAAGCCA
GAS5 siRNA-2 anti-sense	TGGCTTGAGTTAGGCTTGC
GAS5 shRNA sense	ccggGGACCAGCTTAATGGTTCTttcaagagaAGAACCATTAAGCTGGTCCtttttg
GAS5 shRNA anti-sense	aattcaaaaaGGACCAGCTTAATGGTTCTtctcttgaaAGAACCATTAAGCTGGTCC
EZH2 shRNA sense	ccggCGGAAATCTTAAACCAAGAATctcgagATTCTTGGTTTAAGATTTCCGttttt
EZH2 shRNA anti-sense	aattcaaaaaCGGAAATCTTAAACCAAGAATctcgagATTCTTGGTTTAAGATTTCCG
E2F4 siRNA-1 sense	GCGGCGGAUUUACGACAUUTT
E2F4 siRNA-1 anti-sense	AAUGUCGUAAAUCCGCCGCTT
E2F4 siRNA-2 sense	GCCACCACCUGAAGAUUUGTT
E2F4 siRNA-2 anti-sense	CAAAUCUUCAGGUGGUGGCTT
E2F4 siRNA-3 sense	GGAAGCCUCACGUCCAAAUTT
E2F4-siRNA-3 anti-sense	AUUUGGACGUGAGGCUUCCTT

### Gene knockdown

Small interfering RNA (siRNA) and short hairpin RNA (shRNA) were used for knockdown of genes. GAS5 siRNAs, E2F4 siRNAs and the control siRNAs were synthesized by RiboBio (RiboBio Co., Ltd., Guangzhou, China), and the sequences are list in Table 2. The annealed oligos of GAS5 shRNA (sense: 5′-ccggGGACCAGCTTAATGGTTCTttcaagagaAGAACCATTAAGCTGGTCCtttttg-3′, anti-sense: 5′-aattcaaaaaGGACCAGCTTAATGGTTCTtctcttgaaAGAACCATTAAGCTGGTCC-3′) were inserted into GV122 vector, digested with AgeI and EcoRI (Genechem, Shanghai Genechem Co,. Ltd., Shanghai, China). EZH2 shRNA-1, -2 and the nontarget shRNA plasmids were presented from Dr. Bai^49^. The siRNAs and shRNAs were transfected into cells with Lipofectamine 2000 (Invitrogen).

### Gene overexpression

Full-length GAS5 sequence lacking a poly A tail was synthesized (based on the GAS5 sequence, NR_002578.2) and subcloned into a GV144 vector (XhoI and BamHI digestion) (Genechem). Full-length EZH2 sequence lacking a poly A tail was synthesized and subcloned into a PC3.1 vector, and that is a gift from Dr. Qiangsong Tong (Union Hospital, Wuhan, China). The plasmids were transfected into cells as previously described^50^.

### Cell viability assay

3-[4,5-Dimethylthiazol-2-yl]-2,5-diphenyltetrazolium bromide (MTT; Sigma, St. Louis, MO) was used to detect cell proliferation, and cell viability assay was performed as previously described^14^.

### Flow cytometric analysis

Apoptosis and necrosis were assessed by flow cytometry following Phycoerythrin (PE)-conjugated Annexin V (PE Annexin V) and 7-Amino-Actinomycin D (7AAD) staining (BD Pharmingen, San Diego, CA, USA). Cells were treated according to the manufacturer’s instructions and analyzed by flow cytometry (FACScan, Becton Dickinson, Mountain View, CA, USA). Data analysis was performed using Cell Quest analysis software (Becton Dickinson) and expressed as percentage of positive cells.

### Western-blot analysis

The procedure was described previously^50^. Briefly, equal amounts (30-60 μg) of total protein were separated by SDS-PAGE, followed with alternative immunoblot analysis with antibodies [mouse anti-human GAPDH (Cat No. #51332, Cell Signaling Technology, Danvers, MA, USA), mouse anti-human caspase-3 and cleaved caspase-3 (Cat No. #9668, Cell Signaling Technology), rabbit anti-human EZH2 (Cat No. #5246, Cell Signaling Technology), rabbit anti-human E2F4 (Cat No. sc-1082, Santa Cruz Biotechnology, Dallas, Texas, USA), rabbit anti-human GST (Cat No. sc80998, Santa Cruz Biotechnology)]. Immunoreactive bands were then visualized, and the optical densities were measured.

### Quantification of miRNA expression

The levels of mature miR-101 were measured by Bulge-Loop miRNAs qPCR Primer Set (RiboBio Co., Ltd., Guangzhou, China). After cDNA was synthesized with a miRNA-specific stem-loop primer, qPCR was performed with the specific primers^14^. The relative miRNA levels were normalized to those of U6 (RiboBio Co., Ltd.,) small nuclear RNA.

### Luciferase reporter assay

Human EZH2 luciferase reporter plasmid was previously prepared by our team^19^, and human miR-101-2 reporter plasmid was a gift from Dr. Angang Yang^43^. Firefly and Renilla luciferase activities were measured consecutively 40–44 h after transfection, according to the Dual-Luciferase Assay instruction (Promega, Shanghai, China). The activity of Firefly luciferase was normalized by Renilla luciferase.

### RNA immunoprecipitation (RIP)

Cells were harvested and then lysed by nuclear isolation buffer (1.28 M sucrose, 40 mM Tris-HCl, 20 mM MgCl_2_, 4% Triton X-100). Nuclei were pelleted by centrifugation at 2500 g for 15 min, and nuclear pellet was resuspended in RIP buffer (150 mM KCl, 25 mM Tris, 5 mM EDTA, 0.5 mM DTT, 0.5% NP-40, Protease Inhibitor, 100 U/ml RNase inhibitor). Next, resuspended nuclei were split into three fractions (for Input, IgG and, E2F4 antibody coprecipitation) and sheared by a dounce homogenizer. Then, nuclear membrane and debris were pelleted by centrifugation at 13,000 rpm for 10 min. IgG (Santa Cruz Biotechnology) or human anti-E2F4 antibody (Santa Cruz Biotechnology) was added to supernatant with incubation at 4 °C for 2 h. Followed, 40 μl of protein A beads were added and incubated for 1 h at 4 °C. After centrifugation for 30 s, three washes in 500 μl RIP buffer and one wash in PBS, beads were resuspended in 1 ml Trizol. Finally, coprecipitated RNAs were isolated and GAS5 was quantified by RT-qPCR.

### Chromatin immunoprecipitation (ChIP)

The ChIP assay was performed according to the manufacturer’s protocol using the EZ-ChIP kit (Upstate Biotechnology, Lake Placid, NY, USA). Purified DNA was then used for RT-qPCR assay. The primers were designed by the Premier Primer 5.0 software (GAPDH: forward, 5′-GAAGGTGAAGGTCGGAGTC-3′, reverse, 5′-GAAGATGGTGATGGGATTTC-3′; EZH2 promoter: forward, 5′-ACTTGGCTTCCAGCACCCG-3′, Reverse, 5′- CGCTGTAAGGGACGCCACTG-3′).

### RNA pull-down assay

Biotin-labeled RNA pull-down was performed as described previously^51^. Briefly, cellular nuclear protein was extracted by using the NE-PER Nuclear and Cytoplasmic Extraction Reagents (Thermo Fisher Scientific, Inc., USA), and then incubated with biotin-labeled GAS5 truncation probe and streptavidin agarose beads (Invitrogen). Finally, the retrieved protein was detected by Western-blot.

### GST-E2F4 fusion protein

To verify the direct reaction between GAS5 and E2F4, the GST-E2F4 fusion protein was synthesized. Briefly, the E2F4 gene PCR product and the pGEX-6p-1 vector were both digested by EcoRI and BamHI enzymes and then ligated together. Next, the pGEX-6p-1-E2F4 plasmid was transformed into BL21 (DE3) bacteria (Life Technologies, Waltham, MA, USA). The OD600 was periodically measured until it reached 0.6–0.8, and the culture was induced with 0.5 mM IPTG for 5 h at 37 ℃. Soluble lysate was purified in batches by using Glutathione Sepharose 4B beads (Cat No. 17075601, GE Healthcare) for 5 h at 4 ℃. The beads were washed three times with binding buffer, and the fusion protein was separated by SDS-PAGE and identified by Western-blot.

### In vivo experiments

Four-week-old Balb-c nude mice were housed and fed as previously described^14^. The mice were randomly divided into two groups, and EJ cells (1 × 10^6^) with nontarget shRNA or GAS5 shRNA stable transfection were injected s.c. respectively. Two weeks later, each group was randomly divided into two groups, and GA (1.8 mg kg^−1^) or the vehicle was administered i.v. to mice once every other day. GA in that dose were found to be well tolerated by mice in our preliminary experiments. Sixteen days after that, the mice were sacrificed and the tumors were removed, photographed and weighed. Additionally, tumor tissues were collected for RT-qPCR, Western-blot, and immunohistochemistry.

### Immunohistochemistry

Immunostaining was performed on mice tissue sections that had been confirmed as bladder cancer beforehand by a pathologist. The sections were deparaffinized in xylene, rehydrated with ethanol, and then blocked with 3% H_2_O_2_ followed by blocking in goat serum and incubation with anti-EZH2 antibody overnight at 4 °C. Secondary staining was performed and colorized using Vectastain ABC Kit (Vector Laboratories, Burlingame, CA, USA). The peroxidase reaction was developed with diaminobenzidine (DAB kit; Vector Laboratories) and the slides were counterstained with hematoxylin. The images were observed using a microscope (Olympus BX60, Tokyo, Japan) and quantitatively analyzed by Image-Pro Plus software (Media Cybernetics, Carlsbad, CA, USA).

### Statistical analysis

Statistical analysis was performed using SPSS 19.0 software (SPSS Inc, Chicago, IL, USA). The χ^2^ test was used to evaluate the difference in clinical pathological features. Paired t test was performed to compare the difference between paired tissues by RT-qPCR analysis. The Student t test was utilized to compare continuous variables between different groups, summarized as means ± SD of triplicates. *P* < 0.05 was considered significant statistically difference.

## References

[1] Hedegaard J (2016). Comprehensive transcriptional analysis of early-stage urothelial carcinoma. Cancer Cell..

[2] Babjuk M (2013). EAU guidelines on non-muscle-invasive urothelial carcinoma of the bladder: update 2013. Eur. Urol..

[3] Isin M, Dalay N (2015). LncRNAs and neoplasia. Clin. Chim. Acta.

[4] Lü MH (2016). Long noncoding RNA BC032469, a novel competing endogenous RNA, upregulates hTERT expression by sponging miR-1207-5p and promotes proliferation in gastric cancer. Oncogene.

[5] Wang M (2015). Long non-coding RNA MEG3 induces renal cell carcinoma cells apoptosis by activating the mitochondrial pathway. J. Huazhong Univ. Sci. Technol. [Med. Sci.].

[6] Mourtada-Maarabouni M, Pickard MR, Hedge VL, Farzaneh F, Williams GT (2008). GAS5, a non-protein-coding RNA, controls apoptosis and is downregulated in breast cancer. Oncogene.

[7] Wang TH (2017). 2-O-Methylmagnolol upregulates the long non-coding RNA, GAS5, and enhances apoptosis in skin cancer cells. Cell Death Dis..

[8] Li W (2016). Downregulation of LncRNA GAS5 causes trastuzumab resistance in breast cancer. Oncotarget.

[9] Liu Z (2013). Downregulation of GAS5 promotes bladder cancer cell proliferation, partly by regulating CDK6. PLoS ONE.

[10] Zhang H, Guo Y, Song Y, Shang C (2016). Long noncoding RNA GAS5 inhibits malignant proliferation and chemotherapy resistance to doxorubicin in bladder transitional cell carcinoma. Cancer Chemother. Pharmacol..

[11] Gupta RA (2010). Long non-coding RNA HOTAIR reprograms chromatin state to promote cancer metastasis. Nature.

[12] Li CH (2016). EZH2 coupled with HOTAIR to silence microRNA-34a by the induction of heterochromatin formation in Human Pancreatic Ductal Adenocarcinoma. Int. J. Cancer.

[13] Chen WM (2016). Long intergenic non-coding RNA 00152 promotes tumor cell cycle progression by binding to EZH2 and repressing p15 and p21 in gastric cancer. Oncotarget.

[14] Wang Y (2014). Methyl jasmonate sensitizes human bladder cancer cells to gambogic acid-induced apoptosis through down-regulation of EZH2 expression by miR-101. Br. J. Pharmacol..

[15] Luo M (2013). Long non-coding RNA H19 increases bladder cancer metastasis by associating with EZH2 and inhibiting E-cadherin expression. Cancer Lett..

[16] Zhang Y (2017). An androgen reduced transcript of LncRNA GAS5 promoted prostate cancer proliferation. PLoS ONE.

[17] Ishaq M (2016). Functional inhibition of Hsp70 by Pifithrin-μ switches gambogic acid induced caspase dependent cell death to caspase independent cell death in human bladder cancer cells. Biochim. Biophys. Acta.

[18] Ishaq M (2014). Gambogic acid induced oxidative stress dependent caspase activation regulates both apoptosis and autophagy by targeting various key molecules (NF-κB, Beclin-1, p62 and NBR1) in human bladder cancer cells. Biochim. Biophys. Acta.

[19] Wu X (2016). BRD4 regulates EZH2 transcription through upregulation of C-MYC and represents a novel therapeutic target in bladder cancer. Mol. Cancer Ther..

[20] Santos M (2014). In vivo disruption of an Rb-E2F-Ezh2 signaling loop causes bladder cancer. Cancer Res..

[21] Yamaguchi H, Hung MC (2014). Regulation and role of EZH2 in cancer. Cancer Res. Treat..

[22] Bracken AP (2003). EZH2 is downstream of the pRB-E2F pathway, essential for proliferation and amplified in cancer. EMBO J..

[23] Parisi T, Bronson RT, Lees JA (2009). Inhibition of pituitary tumors in Rb mutant chimeras through E2f4 loss reveals a key suppressive role for the pRB/E2F pathway in urothelium and ganglionic carcinogenesis. Oncogene.

[24] Friedman JM (2009). The putative tumor suppressor microRNA-101 modulates the cancer epigenome by repressing the polycomb group protein EZH2. Cancer Res..

[25] Wang L (2014). c-Myc-mediated epigenetic silencing of MicroRNA-101 contributes to dysregulation of multiple pathways in hepatocellular carcinoma. Hepatology.

[26] Martens-Uzunova ES (2014). Long noncoding RNA in prostate, bladder, and kidney cancer. Eur. Urol..

[27] Elkin M (1995). The expression of the imprinted H19 and IGF‐2 genes in human bladder carcinoma. FEBS Lett..

[28] Ying L (2013). Downregulated MEG3 activates autophagy and increases cell proliferation in bladder cancer. Mol. Biosyst..

[29] Zhou Y, Zhang X, Klibanski A (2012). MEG3 noncoding RNA: a tumor suppressor. J. Mol. Endocrinol..

[30] Cao Q, Ning W, Juan QI, Zhengqin GU, Shen H (2016). Long non-coding RNA-GAS5 acts as a tumor suppressor in bladder transitional cell carcinoma via regulation of chemokine (C-C motif) ligand 1 expression. Mol. Med. Rep..

[31] Hung T (2011). Extensive and coordinated transcription of noncoding RNAs within cell-cycle promoters. Nat. Genet..

[32] Zhu J (2015). Long noncoding RNA MEG3 interacts with p53 protein and regulates partial p53 target genes in hepatoma cells. PLoS ONE.

[33] Kino T, Hurt DE, Ichijo T, Nader N, Chrousos GP (2010). Noncoding RNA gas5 is a growth arrest- and starvation-associated repressor of the glucocorticoid receptor. Sci. Signal..

[34] Sun M, Gadad SS, Kim DS, Kraus WL (2015). Discovery, annotation, and functional analysis of long noncoding RNAs controlling cell-cycle gene expression and proliferation in breast cancer cells. Mol. Cell.

[35] Zhan L (2014). Promising roles of mammalian E2Fs in hepatocellular carcinoma. Cell Signal..

[36] Luo G (2017). LncRNA GAS5 inhibits cellular proliferation by targeting P27(Kip1). Mol. Cancer Res..

[37] Kim KH, Roberts CWM (2016). Targeting EZH2 in cancer. Nat. Med..

[38] Christofides A, Karantanos T, Bardhan K, Boussiotis VA (2016). Epigenetic regulation of cancer biology and anti-tumor immunity by EZH2. Oncotarget.

[39] Lee SR (2015). Activation of EZH2 and SUZ12 regulated by E2F1 predicts the disease progression and aggressive characteristics of bladder cancer. Clin. Cancer Res..

[40] Coe BP (2013). Genomic deregulation of the E2F/Rb pathway leads to activation of the oncogene EZH2 in small cell lung cancer. PLoS ONE.

[41] Dimova DK, Dyson NJ (2005). The E2F transcriptional network: old acquaintances with new faces. Oncogene.

[42] Kottakis F (2011). FGF-2 Regulates cell proliferation, migration, and angiogenesis through an NDY1/KDM2B-miR-101-EZH2 pathway. Mol. Cell.

[43] Wang L (2014). c-Myc-mediated epigenetic silencing of MicroRNA‐101 contributes to dysregulation of multiple pathways in hepatocellular carcinoma. Hepatology.

[44] Martínezfernández M (2015). EZH2 in bladder cancer, a promising therapeutic target. Int. J. Mol. Sci..

[45] Zhao L, Guo QL, You QD, Wu ZQ, Gu HY (2004). Gambogic acid induces apoptosis and regulates expressions of Bax and Bcl-2 protein in human gastric carcinoma MGC-803 cells. Biol. Pharm. Bull..

[46] Zhao K (2017). Gambogic acid suppresses cancer invasion and migration by inhibiting TGFbeta1-induced epithelial-to-mesenchymal transition. Oncotarget.

[47] Shahabipour F (2017). Naturally occurring anti-cancer agents targeting EZH2. Cancer Lett..

[48] Wang X (2011). Studies on chemical modification and biology of a natural product, gambogic acid (III): determination of the essential pharmacophore for biological activity. Eur. J. Med. Chem..

[49] Bai J (2015). Enhancer of zeste homolog 2 depletion induces cellular* s*enescence via histone demethylation along the INK4/ARF locus. Int. J. Biochem. Cell Biol..

[50] Xiang W (2015). miR-106b-5p targets tumor suppressor gene SETD2 to inactive its function in clear cell renal cell carcinoma. Oncotarget.

[51] Xiang JF (2014). Human colorectal cancer-specific CCAT1-L lncRNA regulates long-range chromatin interactions at the MYC locus. Cell Res..

